# 3D-Printed Contact Lenses to Release Polyvinyl Alcohol as a Therapeutic Agent for the Treatment of Dry Eyes

**DOI:** 10.3390/pharmaceutics17020219

**Published:** 2025-02-08

**Authors:** Piyush Garg, Parvin Shokrollahi, Haile Fentahun Darge, Chau-Minh Phan, Lyndon Jones

**Affiliations:** 1 Centre for Ocular Research & Education (CORE), School of Optometry & Vision Science, University of Waterloo, 200 University Avenue West, Waterloo, ON N2L 3G1, Canada; p24garg@uwaterloo.ca (P.G.); p.shokrollahi@uwaterloo.ca (P.S.); haile.darge@uwaterloo.ca (H.F.D.); chau-minh.phan@uwaterloo.ca (C.-M.P.); 2 Centre for Eye and Vision Research (CEVR), 17W Hong Kong Science Park, Hong Kong

**Keywords:** three-dimensional-printed contact lenses, dry eye disease, polyvinyl alcohol, shape fidelity, desiccation stress

## Abstract

**Purpose:** Dry eye disease is highly prevalent, and the most common treatment, lubricating eye drops, only remains effective for a very short period of time. This project aims to 3D print a proof-of-concept, custom-fit, polyvinyl alcohol (PVA)-eluting contact lens (CL) for the treatment of dry eye disease. PVA is a commonly used viscosity enhancer in eye drops, with the capability of reducing symptoms of dry eye by stabilizing the tear film and reducing tear evaporation. The protective effects of PVA could be attributed to its water-retaining ability, which provides moisturization and prevents the loss of water. **Method:** In this work, a low-cost stereolithography-based 3D printer was retrofitted with a humidity and temperature control kit to 3D print a PVA-loaded custom-fit CL. To evaluate the print quality of the 3D-printed CL, circularity was used to evaluate the shape fidelity in 3D printing. The PVA release from these lenses was assessed, along with its role in acting as a viscosity enhancer. The effect of PVA was further analyzed by a dry eye disease (desiccation stress) cell model. **Results:** The shape fidelity evaluation of the 3D-printed CL displayed excellent circularity. The diameter, sagittal depth, and base curve of the 3D-printed lenses were measured to be 14.27 ± 0.06 mm, 3.77 ± 0.16 mm, and 6.4 ± 0.24 mm, respectively. The PVA release curves showed that approximately 1300 µg of PVA was released over the study duration of 24 h. **Conclusions:** Overall, this work demonstrates that a 3D-printed PVA-eluting CL is a promising candidate for the treatment of dry eye.

## 1. Introduction

There has been significant interest in using contact lenses (CLs) as drug delivery devices due to their hypothesized ability to increase the residence time of drugs on the ocular surface. However, CL wearers frequently complain of CL discomfort, often associated with dry eye disease, which sometimes results in the discontinuation of lens wear [1]. The placement of a CL onto the ocular surface disrupts the structure and stability of the tear film by dividing it into a pre-lens and post-lens tear film [2]. A strategy to treat dry eyes associated with CL discomfort involves the topical delivery of various ocular lubricants, such as polyvinylpyrrolidone, hyaluronic acid, hydroxypropyl methylcellulose, and polyvinyl alcohol (PVA), via a CL [2]. However, to date, only one PVA-releasing CL material, nelfilcon A (Alcon, Ft Worth, TX, USA), is commercially available; it has been shown to stabilize the tear film and improve eye comfort [3]. This CL’s material releases PVA from the lens through simple diffusion for a short period of time upto one hour at a relatively low concentration [3,4]. Additionally, the blister pack solution of this lens contains other moisturizing agents, including hydroxypropyl methylcellulose, polyethylene glycol, and PVA, which are released from the lens when in situ due to the natural blinking action of the eye [3,5]. The concept of releasing lubricating agents onto the ocular surface using a CL offers several advantages, including protecting the surface of the eyes by keeping them moist and enhanced patient compliance compared with frequent instillation of ocular lubricants.

The majority of CLs are mass-fabricated using molding methods which produce CLs on a large scale. However, this method is unsuitable for customization to meet the needs of an individual patient, an approach towards so-called ‘personalized medicine’. This customization could be related to patient-specific fitting factors (especially related to diameter or base curve radius), as well as factors related to various drug concentrations, depending on the individual ocular condition being treated. Another issue is that the storage conditions of a drug-eluting CL may have an impact on drug stability and hence on the release profiles of drugs and therapeutic molecules from these lenses. This could lead to differences in the amount and rate of drug released depending on when the drug-eluting CL was manufactured. Therefore, a method to fabricate personalized CLs for an individual patient as they are needed (inspired by so-called ‘just-in-time’ concepts) would be helpful.

Recent advancements in three-dimensional (3D) printing are unlocking new possibilities for the development of CL tailored for medical applications, including ocular drug delivery. The ability to design complex and personalized objects from a wide range of materials makes 3D printing an ideal method for creating customized CLs with precise therapeutic properties. Three-dimensional printing, a collective term for additive manufacturing techniques, involves building objects layer-by-layer from digital models using various materials [6]. Several 3D printing technologies have been explored to fabricate these specialized CLs, including fused deposition modeling (FDM), selective laser sintering (SLS), stereolithography (SLA), and digital light processing (DLP) [7]. These methods offer distinct advantages for producing lenses with integrated drug delivery systems or advanced features to address specific medical conditions, such as vision disorders. For instance, vat photopolymerization 3D printing offered a novel approach to functionalizing commercial CL by modifying existing lenses rather than printing entire curved structures. By printing on a flat lens attached to a build plate, the technique avoided complexities and allowed the lens to regain its original shape post-printing. This approach was shown to be compatible with commercial printers, enabling multi-material deposition, and facilitated the addition of functionalities like color blindness correction or drug delivery, simplifying the process and expanding potential applications in CL technology [8].

In another study, 3D printing was leveraged to address color blindness by enabling the precise deposition of color-filtering dyes, such as Atto 565 and 488, into polyHEMA resin during lens fabrication. This allowed for the creation of specific filtering regions targeting problematic wavelengths and for customization by tailoring the dye concentration and distribution to individual needs, optimizing color perception [9]. For a similar application, another study introduced a multi-material 3D printing approach that enabled the fabrication of CLs with varying material properties within a single structure, addressing the limitations of traditional lenses. The approach allowed for targeted functionality, such as incorporating specific dyes to filter wavelengths of light for color blindness correction, which was difficult with uniform compositions. It simplified manufacturing by integrating multiple functionalities into a single printing step and enabled personalized solutions by tailoring material placement and properties to individual needs [10].

3D printing for ocular drug delivery, specifically vat photopolymerization, facilitated the fabrication of a drug-eluting CL by enabling precise azithromycin loading within a PEGDA hydrogel, ensuring uniform distribution and controlled release [9]. However, despite these advantages, the 3D printing of CLs does have some limitations. The step effect, a staircase-like surface irregularity inherent to the layer-by-layer nature of 3D printing, poses challenges for CLs, where smoothness is critical for comfort and optical performance. This issue was addressed using a ‘continuous liquid film confined 3D printing strategy,’ where a thin resin film adhered to the cured structure during printing, filling gaps between layers to smooth the surface. By carefully controlling the film’s thickness and viscosity through resin and printing parameters, nanoscale surface roughness was achieved, eliminating the need for post-washing and resulting in a smooth surface in the CL [11].

This work explores an on-demand 3D printing or additive manufacturing technique as an alternate method for fabricating custom-fit CLs on a small scale [12]. Moreover, 3D printing offers several advantages, such as precise control over object dimensions using computer-aided design models, custom fit, manufacturing of multiple lenses at the same time for an individual patient, and minimal post-processing [13]. SLA was chosen in this study due to its high resolution and reasonably smooth surface quality, features that are critical for achieving optical clarity and comfortable CL wear. Its compatibility with biocompatible resins, such as GelMA, enables the fabrication of hydrophilic and transparent lenses, suitable for ocular applications. SLA also facilitates the precise incorporation of functional materials, supports complex geometries and customization, and streamlines production through fast fabrication cycles and minimal post-processing, making it an effective choice for developing functional CLs [6]. Owing to the precision and high resolution required for the 3D printing of medical devices, SLA-based techniques may prove to be superior compared to other extrusion-based printing methods, which are limited by low-viscosity materials [14]. SLA-based 3D printing makes it possible to use different photopolymerizable materials loaded with therapeutics of interest to fabricate CL with desired functionalities. Moreover, this technique offers freedom of design fabrication, higher resolution, and improved shape fidelity [12]. The print quality and shape fidelity of these 3D-printed CLs can be described using circularity. Shape fidelity refers to the accuracy with which the 3D-printed object matches the intended original computer-aided design (CAD) or model, and circularity refers to the precision of the shape printed [15]. Although the process of 3D printing can lead to a staircase effect (concentric rings) on the surface, there are techniques available to improve surface smoothness to address the staircase effect, such as decreasing layer thickness while 3D printing. An ideal post-processing method should have lower labor and material costs along with higher consistency; however, such methods lead to higher fabrication times and equipment costs. Recently, Xue et al. proposed a post-processing method in which the printed structure was soaked in a resin tank after the print was completed, following which the print plate was rotated vertically upwards for the extrusion printing mode and the liquid resin formed on the surface was cured with the help of UV light. These ‘additive’ methods, where a layer of resin is formed on the surface owing to surface tension, could be useful to address the issue of the staircase effect on 3D-printed gelatin methacrylate (GelMA)-based soft materials. The surface altitude of printed lenses was measured using a 3D profiler and the authors reported that based on this printing method, with high shape accuracy and thickness resolution, these lenses could control light refraction [16].

In terms of mechanism of action, lubricating agents released from 3D-printed custom fit lenses can act as viscosity-building macromolecules to form a protective layer over the ocular surface, thereby reducing the desiccation stress experienced by the cornea in a dry eye situation. PVA is a commonly used viscosity enhancer in eye drops [17], with the capability of reducing the symptoms of dry eye by stabilizing the tear film and reducing evaporation. Ocular lubricants to treat moderate dry eye that contain PVA include Blink™ (Johnson & Johnson, Jacksonville, FL, USA), Refresh Classic™ (Allergan, Dublin, Ireland), and Liquifilm™ (Allergan, Dublin, Ireland). These lubricating eye drops all contain approximately 1.4% PVA [4].

In this work, an SLA-based technique was used to 3D print a custom-fit CL which can release PVA as a viscosity-enhancing agent for potential applications in dry eye. This work shows that the printing parameters can be optimized to achieve successful 3D printing using a low-cost commercially available 3D printer. To evaluate how closely a 3D-printed CL resembles the computer-aided design model, circularity was used to evaluate the shape fidelity in 3D printing. Next, GelMA-PVA hydrogels were prepared and printed as PVA-releasing CLs for dry eyes. As previously discussed, there is a commercially available CL material that releases PVA. However, this CL material only releases non-polymerized PVA for a short period of time at a very low concentration [3,4]. In this manuscript, we report on the incorporation of PVA into a CL structure that can extend the release period up to 24 h. The PVA release from these lenses was studied along with its ability to protect the ocular surface against desiccation stress using a dry eye disease model.

## 2. Materials and Methods

### 2.1. Synthesis of GelMA

GelMA was prepared by the direct reaction of 40 g Type A gelatin (Sigma, St. Louis, MO, USA) with methacrylic anhydride (1% *v*/*v*, Sigma, St. Louis, MO, USA) in 400 mL carbonate bicarbonate buffer (CB Buffer, 12.5 mM sodium bicarbonate, 87.5 mM sodium carbonate anhydrous, pH 9.4) at 50 °C for 1 h. The substitution reaction was stopped by adding 6 mL of 10% acetic acid (Sigma, St. Louis, MO, USA) and the resulting product was dialyzed in 12–14 kDa dialysis tubes (Sigma, St. Louis, MO, USA) in deionized water for 24 h to remove low molecular weight impurities. After dialysis, the GelMA solution was freeze-dried (Labconco Freeze-Dry System FreeZone 2.5 Liter, 7670000, Kansas City, MO, USA) and stored at −80 °C until further use.

### 2.2. Preparation of GelMA and P-Gel Inks

To prepare the GelMA ink, lithium phenyl-2,4,6-trimethylbenzoylphosphinate (0.6% *w*/*v*, LAP, Sigma, St. Louis, MO, USA) solution was made in phosphate-buffered saline (PBS, pH 7.4, Fisher Scientific, Waltham, MA, USA) and freeze-dried GelMA was added to it to give 10% (*w*/*v*) solution. To make PVA/GelMA (P-Gel-3%) ink, GelMA (20% *w*/*v*) was added to LAP (1.2% *w*/*v*) solution dissolved in PBS. PVA (6% *w*/*v*, molecular weight 89–98 kDa, 99+% hydrolyzed, Sigma, St. Louis, MO, USA) solution was made separately in PBS and heated up to 90 °C until completely dissolved. The two solutions were added in a 1:1 ratio to make the P-Gel-3% ink comprising GelMA (10% *w*/*v*), PVA (3% *w*/*v*), and LAP (0.6% *w*/*v*). A similar method was used to make P-Gel-1.5% ink. These solutions were incubated at 37 °C for 1 h before use.

### 2.3. Three-Dimensional Printing of Contact Lenses

**Scheme 1:**
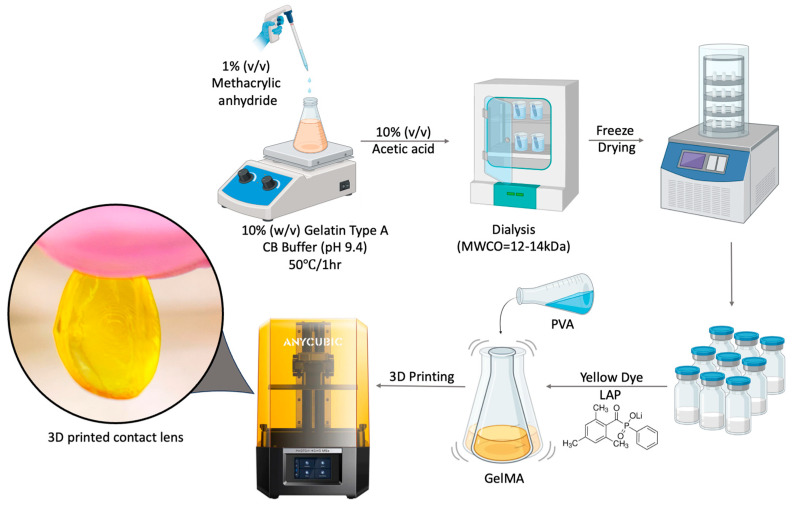
Scheme of preparation of P-Gel prepolymer solution and 3D printing of CL.

Contact lenses with a diameter of 14.98 mm and a height of 3.6 mm were designed using a computer-aided design software (FreeCAD 0.19) and then 3D-printed using a commercial masked stereolithography (mSLA) 3D printer (AnyCubic Photon Mono M5, Shenzhen, Guangdong, China). The printer was retrofitted with a humidity and temperature control kit to enable printing at ~70% humidity and 37 °C. The GelMA and P-Gel ink solutions were incubated at 37 °C for 1 h and mixed gently before use. The 3D printing photopolymerization process was carried out using UV light of wavelength 405 nm. Scheme 1 depicts the stages of preparation of the P-Gel polymer solution and 3D printing of the CL. The exposure time for the layers was set to 80 s, while the first 3 base layers were exposed to 120 s of UV light. The layer thickness while printing was set to 100 µm. These 3D-printed lenses were washed with warm PBS to remove any uncured polymer solution and were post-cured with UV light (405 nm).

### 2.4. Print Quantification

The 3D-printed CLs were imaged using a mobile phone (Galaxy S23, Samsung Electronics, Suwon, Republic of Korea) and were analyzed using Fiji Software (Version 2.14.0/1.54f)￼ [18]. Using a 2D top view, the images were analyzed and two key parameters of 3D printing, shape fidelity and circularity, were calculated. Shape fidelity was assessed by determining the ratio between the experimental printed area (*A_p_*) and the theoretical area (*A_t_*), as well as the ratio between the experimental printed circumference (*C_p_*) and the theoretical circumference (*C_t_*). For a perfect circle, the shape fidelity scores should be equal to 1. For circular structures, the precision of shape is assessed using a parameter called ‘circularity’ [15]. Circularity was calculated using the equation described in Table 1. For a perfect circle, the circularity value would be 1.

Lens metrology measurements (total diameter and sagittal depth) were determined using the Chiltern Optimec (Optimec Limited, Malvern, UK), and the base curve was calculated with the assumption of a spherical lens design. The sagittal height (sag) and diameter (d) measurements were used to calculate the base curve (bc) using the following formula: bc = [sag^2^ + (d/2)^2^]/(sag × 2). Before measurement, the lens chamber was filled with PBS and the lens was placed on the mantle, and the contours of the magnified lens images were sketched. Each lens was measured twice, with the lens rotated approximately 90° on the mantle for the second measurement. The true lens dimensions were calculated under consideration of the magnification factor of the Chiltern (×0.05896), and the average of both measurements was used for analysis [19].

### 2.5. Light Transmittance

The optical transparency of the samples was determined from their light transmittance (%T) using a UV-Vis spectrometer (SpectraMax M5e, Molecular Devices, San Jose, CA, USA). The samples were prepared by pipetting prepolymer solutions of GelMA and P-Gels on a glass coverslip at a thickness comparable to that of a commercially available CL (80 µm) and cured by a 3D printer using a previously described setup [5]. The samples were placed inside a cuvette with the help of a mold. A commercial CL, nelfilcon A (Alcon, Fort Worth, TX, USA), was fitted on a 3D-printed mold and placed inside the cuvette for control measurements. The absorbance of the samples was measured in the wavelength range of 400–800 nm. For the blank measurement, the absorbance of the glass coverslip was measured from 400 to 800 nm. The formula %T = 100 × 10^(−absorbance)^, derived from the Beer–Lambert Law, was used to calculate the light transmitted. Here, %T represents the percentage of light that passes through the hydrogel sample and reaches the detector on the other side.

### 2.6. PVA Release from 3D-Printed CLs

To study the PVA release from these CLs, they were incubated in glass vials with gentle agitation with 8 mL PBS (pH 7.4) at 35 °C. At each indicated time point, 300 µL of the solution was removed for the detection of PVA and replaced with fresh PBS. For the detection of PVA, a solution of iodine (150 mM potassium iodide, 50 mM diiodine in deionized water, Sigma, St. Louis, MO, USA) and borate (64.7 mM boric acid in deionized water, Sigma, St. Louis, MO, USA) was mixed in a 1:5 ratio of iodine solution/borate solution to generate the PVA detection solution. The solution was gently inverted 4–6 times in a conical tube to ensure even mixing. To measure PVA in solution, 163.6 µL of PVA detection solution was added to 36.4 µL of samples of interest in a 96-well plate and incubated at room temperature for 20 min with gentle shaking. Absorbances were read at 630 nm using an Imaging Multimode Plate Reader (Cytation 5, Agilent BioTek Instruments, Winooski, VT, USA) [20]. The PVA release was quantified using a calibration curve. This curve was plotted for standard solutions ranging from 10 to 250 µg/mL. The R^2^ value for the calibration curve was 0.9997.

### 2.7. Drug Release Kinetics via Mathematical Modeling

To evaluate the kinetic release profiles of the samples over the studied time duration, different mathematical models were studied [21]. These included zero- and first-order release model, the Higuchi release model, and the Korsmeyer–Peppas model. The graphs were plotted as mentioned in Table 2, and the coefficient of determination (*R*^2^) was calculated for each model [22]. Formats are correct. Please remove the underline below t.

### 2.8. Viscosity Measurements

The viscosity measurements for different concentrations of PVA solutions made in PBS (ranging from 0.5%, 0.7%, 1.0%, and 1.4% to 2% *w*/*v*) were carried out on a Viscometer L2.16 (Cambridge Applied System, Boston, MA, USA), which uses the concept of variation in the falling body. In brief, a piston (0.25–5 cP) was suspended in an elongated cavity. The electromagnets in the wall force the piston to move in a reciprocating up-and-down motion. The movement of the piston is inversely related to the viscosity of the fluid. The instrument calculates the viscosity of the sample by measuring the speed at which the piston moves [23].

### 2.9. Simulating Dry Eye Conditions via Desiccation Stress on Human Corneal Epithelial Cells

Human Papilloma virus (HPV)-immortalized human corneal epithelial cells (HCECs), previously gifted by Dr. Maud Gorbet at the University of Waterloo, were used in this study. The cell line was screened for morphological, biochemical, and electrophysiological characteristics by Griffith et al. [24,25] The cells were maintained between passages 3–15 and cultured in DMEM/F12 medium (Sigma, St. Louis, MO, USA) containing 1% FBS and 1% penicillin/streptomycin (pen/strep) at 37 °C, 5% CO_2_, and high humidity (>95%). The medium was replaced every 2–3 days until confluency. HCECs were maintained as described above, and 15 × 10^4^ cells were seeded into 96-well plates (ThermoFisher, Waltham, MA, USA), then incubated overnight at 37 °C with high humidity and 5% CO_2_. After 24 h of incubation, the culture medium was removed from the plate. The cells were immediately treated with 150 µL of the test formulations (PVA solutions made in PBS ranging from 0.5%, 0.7%, 1.0%, 1.4%, and 2% *w*/*v*) and the culture medium control solution. The cells were incubated at 37 °C and 5% CO_2_ for 30 min. The test solutions from the cells were removed and the cells placed in the chamber at 37 °C and 45% RH for 2 min to desiccate the cells. Cell viability was assessed using the MTT assay and the absorbance values were measured at 570 nm using a multimode plate reader (Cytation 5, Agilent BioTek Instruments, Winooski, VT, USA) [26].

### 2.10. Statistical Analysis

Statistical analysis was performed using GraphPad Prism Version 10.0 (GraphPad Software, La Jolla, CA, USA). All data are reported as mean and standard deviation (n = 3) unless otherwise stated. A two-way ANOVA was performed to determine differences across testing groups wherever applicable. Tukey’s multiple comparison tests were used when necessary. In all cases, statistical significance was considered at a *p*-value of <0.05, unless otherwise stated.

## 3. Results

### 3.1. Three-Dimensional Printing of Contact Lenses

**Figure 1:**
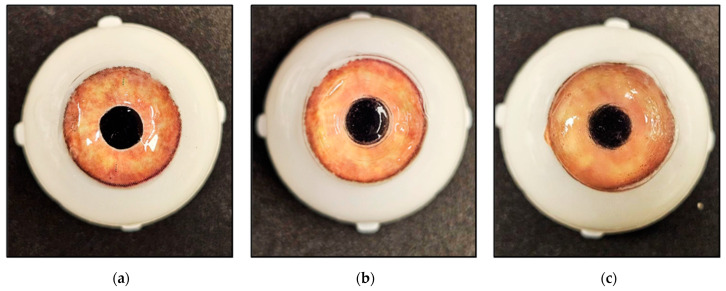
Comparative optical clarity of (**a**) commercially available Dailies Aqua Comfort Plus™ CL and (**b**,**c**) 3D-printed PVA-loaded CLs (P-Gel-1.5% and P-Gel-3%). The images were taken on a 3D-printed eye model mimicking the human eye to show the fitting of these lenses and their optical clarity.

Figure 1 shows the 3D-printed CLs with P-Gel-1.5% and P-Gel-3% inks compared to a commercially available CL (Dailies Aqua Comfort Plus™; Alcon, Fort Worth, TX, USA). The CLs were printed in about an hour and the minimum thickness for the lenses that was reliably printed each time using the current 3D printing setup was 400 µm, which is greater than the typical thickness of a commercial CL (80 µm). It is worth noting that to make these prototype lenses suitable for wearing in a future clinical study, the thickness needs to be reduced. It should be noted that despite the high water content in these lenses, the lenses were successfully 3D-printed with high shape fidelity. These lenses demonstrated clean and sharp edges and could be printed without any support structures. Reports indicate that an effective photocurable biomaterial ink suitable for projection-based printing methods, such as SLA LCD printing, should possess three key characteristics: a fast photo-crosslinking rate, a tightly crosslinked structure, and exceptional fluidity to ensure the swift filling of gaps and spaces [27]. In this work, GelMA was used as a base material owing to its biocompatibility and demonstrated high levels of controllability [28,29]. The concept has been retained.

Another crucial aspect in vat-polymerization is to avoid the evaporation of water during the exothermic polymerization reaction. Consequently, this could lead to an increased viscosity of the printable ink related to the gradual increase in the polymer concentration at the interface and interferes with the layer separation/approach step [30]. To overcome these challenges, 3D printing conditions such as dye concentration and UV exposure time were selected along with proper environment conditions, including temperature (~37 °C) and humidity (~70% RH) [6]. In the 3D printing of hydrogels, dyes are used as photosensitizers to enhance the reactivity of the ink to light and improve the resolution of the printed object by reducing light scattering. Therefore, careful adjustment of the dye concentration is key for effective polymerization and resolution, ensuring successful printing of the hydrogel biomaterials with fine details and higher structural integrity [6]. The results showed that it is possible to 3D print a structure as delicate as a CL with remarkable shape fidelity and accuracy on a low-cost commercial printer.

**Figure 2:**
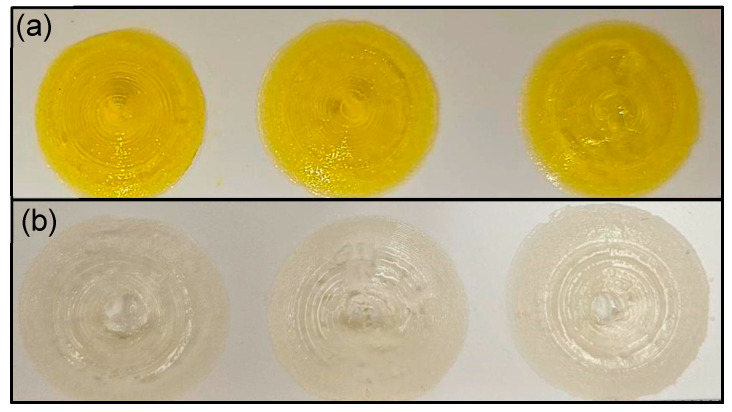
Three-dimensional printed contact lenses (**a**) before and (**b**) after washing and post-curing.

Based on a series of preliminary experiments, the printing window for GelMA concentration (10%), exposure time (80 s), dye concentration (3%), and LAP concentration (0.6%) were optimized and used. The printing window parameters were selected regarding previous reports confirming that an increase in initiator concentration and light intensity can overcome the oxygen inhibition of photopolymerization reactions [31]. The 3D-printed lenses were carefully removed from the print plate and washed in warm PBS to remove any uncured polymer from the surface. Reports suggest that mechanical characteristics such as the elasticity, hardness, and compressibility of GelMA are governed by the kinetics of cross-linking during the photocross-linking process [29]. Therefore, to improve the base mechanical strength of GelMA, several methods are used, such as increasing the prepolymer concentration, higher degree of substitution, and functionalization, or higher UV exposure time [32]. Besides increasing mechanical strength, post-curing also ensures consistency in the final print. Therefore, once the CLs were washed, they were post-cured using a 405 nm UV light. This post-curing step is important to improve the mechanical strength of 3D-printed lenses. Without proper post-curing, the 3D-printed object will be weaker in certain areas, compromising its long-term structural stability. The post-curing step also helps to photobleach the excess yellow dye in these lenses, as shown in Figure 2.

### 3.2. Print Quantification Analysis

The 3D-printed lenses were analyzed for their shape fidelity (area ratio, circumference ratio, and circularity); an ideal score would be equal to 1. The area ratio, circumference ratio, and circularity were calculated based on the formulas described in Table 1.

**Figure 3:**
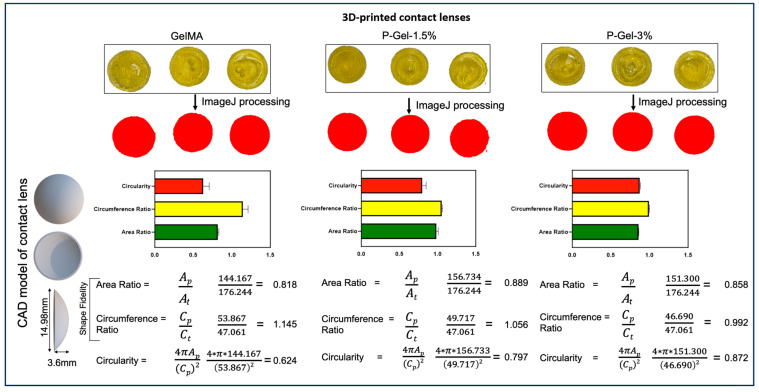
Analysis of shape fidelity and circularity of 3D-printed contact lenses (calculations based on 2D top view).

The area ratios of GelMA, P-Gel-1.5%, and P-Gel-3% were 0.818 ± 0.014, 0.889 ± 0.009, and 0.858 ± 0.004, respectively (Figure 3). The ideal value for area ratio would be equal to 1 and a value of <1 denotes that the printed CL is smaller than the theoretical area and vice versa. Compared to GelMA, the higher area ratios in case of P-Gels could be attributed to the presence of PVA, which makes the structure more robust and mechanically stronger [33,34,35,36]. The circumference ratios of GelMA, P-Gel-1.5%, and P-Gel-3% were 1.145, 1.056, and 0.992, respectively. The ideal value for circumference ratio would also be equal to 1 and a value of >1 or <1 denotes that the printed CL has an irregular boundary. The printed circumference (C*_p_*) calculated using this method was highly variable because even slight irregularities in the periphery of the lenses would contribute largely to the circumference ratio. Hence, an irregularity in the boundary could give a higher false value of printed circumference, even if the print is smaller in area.

The diameter and sagittal depth of the 3D-printed CLs were measured to be 14.27 ± 0.06 mm and 3.77 ± 0.16 mm. The base curve was calculated with the assumption of a spherical lens design. The sagittal depth (sag) and diameter (d) measurements were used to calculate the base curve (bc) using the following formula: bc = [sag^2^ + (d/2)^2^]/(sag × 2) and calculated to be 6.4 ± 0.24 mm [13]. For circular structures, the precision of the shape is assessed using a parameter called ‘circularity’. The circularity of GelMA, P-Gel-1.5%, and P-Gel-3% lenses were 0.624, 0.797, and 0.872, respectively. The ideal values for circularity should be equal to 1. Two lenses based on GelMA or P-Gel inks with the similar perimeter can have different areas if one or both have irregular boundaries, resulting in differing areas. Variations in printing conditions (temperature and humidity), water content, and handling could lead to such changes and, therefore, to a deviation in circularity values. Interestingly, a higher variability (standard deviation) in circularity was observed with the lenses printed with GelMA, which could be attributed to the relatively weaker mechanical properties of GelMA compared to P-Gel. A reduction in variability was observed with the P-Gel CLs owing to their robust and mechanically stronger structures [37,38].

### 3.3. Light Transmittance and Optical Transparency

**Figure 4:**
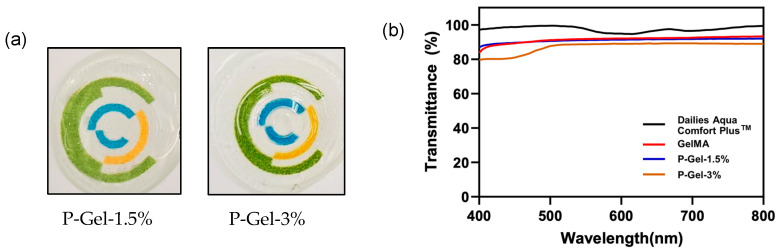
(**a**) Optical transparency of 3D-printed CLs on a colored background. (**b**) Optical transmittance curves of Dailies Aqua Comfort Plus™ (Nelfilcon A), GelMA, P-Gel-1.5%, and P-Gel-3%.

Figure 4a shows the optical clarity of a CL 3D-printed from GelMA and P-Gels containing 1.5% and 3% PVA on a colored background. The transmittance curves for GelMA and P-Gel hydrogels were plotted in the visible range of light at 400–800 nm (Figure 4b). At 450 nm, GelMA gels exhibited about 89.2% transmittance, while P-Gel-1.5% and P-Gel-3% displayed around 89.5% and 80.9% transmittance. At 630 nm, the transmittance of GelMA was 92.2%, and for P-Gels, it was around 91.5% (P-Gel-1.5%) and 89.0% (P-Gel-3%). The mean light transmittance of GelMA, P-Gel-1.5%, and P-Gel-3% at 400–800 nm was about 91.4%, 90.9%, and 87.2%, respectively, which is close to that of a commercially available PVA-containing CL (97.5%) [39]. At 555 nm, the transmittance of GelMA was 91.8%, and for P-Gels, it was around 91.1% (P-Gel-1.5%) and 88.7% (P-Gel-3%). In the visible region (555 nm), daily contact lenses are reported to exhibit an average transmittance between 78% and 82% [40]. The results showed that the transparency decreased with an increase in PVA concentration from 1.5% to 3%, which is likely due to the presence of a high polymeric phase compared to the GelMA hydrogels.

### 3.4. PVA Release

**Figure 5:**
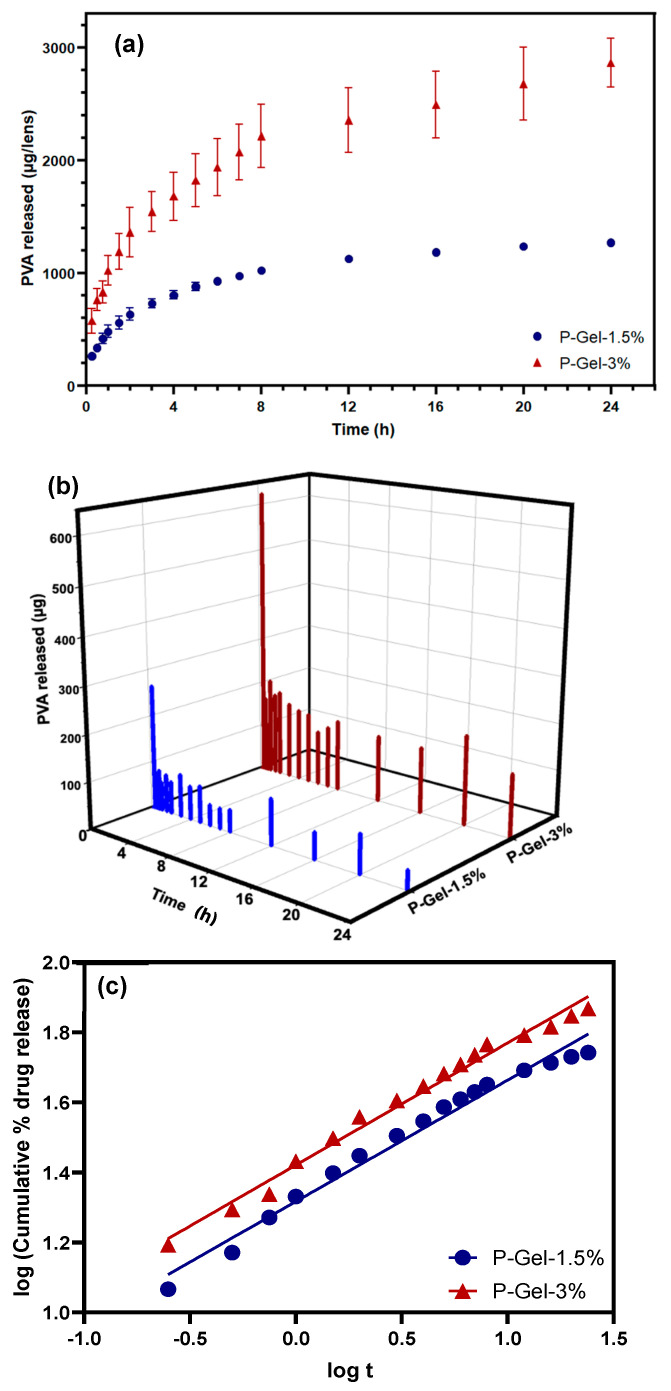
(**a**) Cumulative PVA release curves of P-Gel-1.5% and P-Gel-3%. (**b**) Instantaneous release of PVA (µg/mL) from P-Gel-1.5% and P-Gel-3%. (**c**) Modeling of the PVA release data using the Korsmeyer–Peppas model (*R*^2^ = 0.9806 and *R*^2^ = 0.9864 for P-Gel-1.5% and P-Gel-3%, respectively).

**Figure 6:**
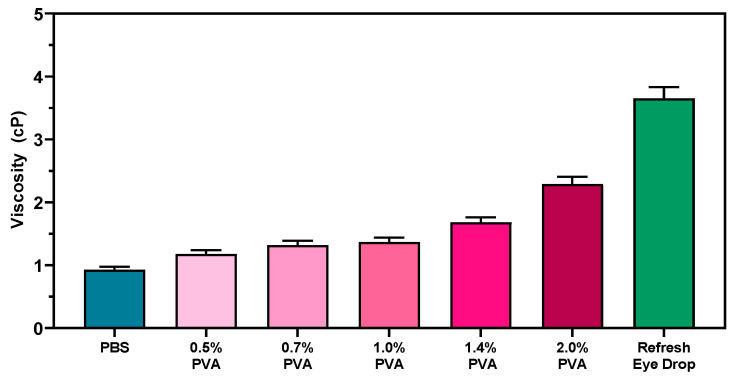
Graph representing viscosity as a function of PVA concentration.

The PVA release curves depict the PVA released from 3D-printed CLs with different PVA concentrations (Figure 5a). About 1300 µg (about 55% of the initial loaded amount) and 2900 µg (about 73% of the initial loaded amount) of PVA was released from P-Gel-1.5% and P-Gel-3% CL at the end of 24 h. The instantaneous release amounts of PVA were calculated (in µg) and plotted as 3D bars with the help of OriginPro 2024 software (Figure 5b). The graph shows that about 40–80 µg PVA was released at every successive time points with P-Gel-1.5%, while for the lenses containing 3% PVA, the release was in the range of 140–180 µg. As the volume of the tear film is about 7–10 µL, the PVA concentration on the ocular surface reaches between 0.5 and 2% (*w*/*v*, as correlated from the viscosity data, Figure 6) [1]. It is anticipated that CLs such as these would be used as daily disposable bandage CLs, for which the typical wear period prior to lens disposal is 12–16 h.

To study the drug release kinetics and how different factors affect the dissolution rate and behaviors, various mathematical equations describing the release dependence on time were used. The drug release was evaluated though four main release kinetic models: zero-order, first-order, the Higuchi model, and the Korsmeyer–Peppas model. To evaluate the best fit model for the release profiles in our study, the goodness of fit for each model or the coefficient of determination (*R*^2^) were calculated using GraphPad Prism (Table 2 and Table 3). The release profiles of the PVA containing hydrogels showed the highest linearity (Figure 5c) with the Korsmeyer–Peppas model (*R*^2^ *=* 0.9806 and *R*^2^ = 0.9864 for P-Gel-1.5% and P-Gel-3%, respectively). The release exponents (*n*) for P-Gel-1.5% and P-Gel-3% samples were 0.3464 and 0.3476, indicating that these systems follow Fickian diffusion (Table 3). In the Fickian model (Case I), drug release is governed by diffusion, and the rate of diffusion is greater than the process of polymeric chain relaxation. The equilibrium of absorption in the surface exposed of the polymeric system takes place rapidly, leading to conditions of time-dependent links. The kinetics of this phenomenon are characterized by diffusivity [41].

### 3.5. Viscosity Measurements

The viscosities of different concentrations of PVA solutions were calculated using a viscometer, and viscosity was plotted as a function of PVS (Figure 6). Ocular lubricants to treat moderate dry eye that contain PVA include Blink™ (Johnson & Johnson, Jacksonville, FL, USA), Refresh Classic™ (Allergan, Dublin, Ireland), and Liquifilm™ (Allergan, Dublin Ireland). These lubricating eye drops all contain approximately 1.4% (*w*/*v*) PVA [4]. Therefore, in this study, the viscosity measurements for different concentrations of PVA solutions made in PBS ranging from 0.5%, 0.7%, 1.0%, and 1.4% to 2% (*w*/*v*) were used. The results demonstrate that viscosity increases with an increase in the amount of PVA and is a function of polymer concentration [42]. The viscosity of 1.4% PVA solution was about 1.72 cP, whereas that of the Refresh Classic eye drop was around 3.65 cP. The active ingredients in Refresh Classic eye drops are 1.4% PVA and 0.6% povidone. Therefore, the higher viscosity of Refresh Classic eye drops at a similar PVA concentration could be due the different composition of PVA in terms of molecular weight (chain number and chain length) [17] and the presence of other agents such as povidone in Refresh Classic eye drops. Viscosity-enhancing agents such as PVA comprise the bulk of artificial tears and have been used in patients with dry eye disease [43]. An ideal viscosity enhancer should possess sufficient viscosity and adequate rheological behavior to lubricate the ocular surface and have sufficient ocular surface residence time. Also referred to as demulcents or lubricants, the US FDA recognizes six categories of these agents that can be used in over-the-counter formulations, which include cellulose derivatives, dextran, gelatin, liquid polyols, polyvinyl alcohol, and povidone [44]. These viscosity-enhancing compounds have hygroscopic properties (water-retaining ability on the ocular surface), allowing them to prevent the loss of water and moisturize the ocular surface [45]. Of note, the compatibility of PVA with the eye compartment is widely known [46] and is a commonly used viscosity enhancer in eye drops [17]. Therefore, 3D-printed custom-fit PVA-eluting CLs could be used as a potential strategy to treat dry eyes.

### 3.6. Ability of PVA to Protect HCECs Against Desiccation Stress

Dry eye disease (DED) is a multifactorial disease with numerous patient symptoms that ultimately lead to discomfort and potential damage to the eye [1]. Although there are several methods to address the various pathophysiological mechanisms behind DED (ranging from stabilizing the tear film to reducing pro-inflammatory responses on the ocular surface) [1], a PVA-releasing CL could be an attractive option. To study the ability of PVA to protect corneal cells against desiccation stress, a previously established protocol was followed [26]. This protocol helps to assess the overall impact of the dry eye formulation on the viability of corneal epithelial cells.

**Figure 7:**
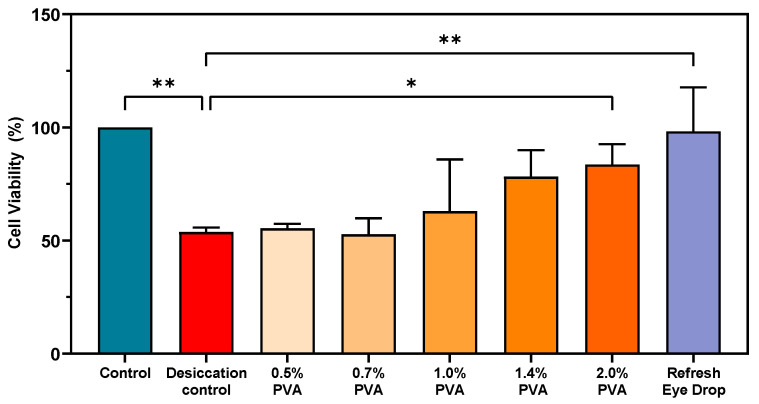
Effect of PVA and Refresh Classic eye drops on HCECs and resistance against desiccation stress (* *p* < 0.05 and ** *p* < 0.005).

Figure 7 demonstrates that PVA at concentrations around 2% showed significantly higher cell viability (*p* < 0.05) compared to the control. Similar results were observed with the Refresh Classic eye drops (*p* < 0.05). Importantly, after the formulation was removed from the culture wells, the chemical interaction between the remaining molecules in contact with the cells impacted the desiccation and provided protection. These results confirm the feasibility of PVA to be loaded in the 3D-printed CLs and protect HCECs against desiccation stress. As indicated from the package contents, Refresh Classic eye drops contain two active ingredients, 1.4% PVA and 0.6% povidone. As previously indicated, the presence of two different active ingredients, as well as different compositions of PVA in terms of molecular weight (chain number and chain length) [17], in Refresh Classic eye drops could be the reason for their higher protection.

## 4. Conclusions

In summary, this work attempts to 3D print a proof-of-concept PVA-eluting CL based on GelMA for potential application in patients with dry eye disease. The PVA-eluting 3D-printed lenses displayed better shape fidelity than GelMA, as indicated by circularity scores of 0.624, 0.797, and 0.872 for GelMA, P-Gel, 1.5%, and P-Gel-3%, respectively. Measurements of the P-Gel-1.5% lenses indicated a diameter of 14.27 ± 0.06 mm and a sagittal depth of 3.77 ± 0.16 mm, which is similar to commercially available lenses. SLA-based 3D printing makes it possible to use different photopolymerizable materials loaded with therapeutics of interest, such as PVA, and to fabricate a CL mimic with desired functionalities. PVA release profiles suggest that approximately 1300 µg of PVA was released over 24 h. PVA is a commonly used viscosity enhancer in eye drops, with the capability of reducing symptoms of dry eye by stabilizing the tear film and reducing evaporation. This was further validated by its ability to protect HCECs against desiccation stress. The mean light transmittance of GelMA, P-Gel-1.5%, and P-Gel-3% at 400–800 nm was 91.4%, 90.9%, and 87.2%, respectively, which is close to a commercially available PVA-containing CL (97.5%). The results of this study suggest that the 3D printing of a delicate structure such as a CL is possible by tuning the material properties.

These lenses could be worn as daily disposable bandage CLs, for which the typical wear period prior to lens disposal is 12–16 h. The current work explores an on-demand 3D printing or additive manufacturing technique as an alternate method for fabricating custom-fit CLs on a small scale. However, another option could be to store these lenses in dry form after manufacturing and rehydrate them in PVA solution to avoid issues related to the premature release of PVA.

Looking forward, we will focus on controlling the release of PVA from gelatin methacrylate-based biomaterials. To convert this material into successful CLs, oxygen transmissibility, fluid and ion permeability, mechanical properties, and lens stiffness need to be optimized.

## Figures and Tables

**Table 1:** Calculations for shape fidelity and circularity of 3D-printed CL.

Shape Fidelity		
$Area\;Ratio=\frac{A_{p}}{A_{t}}$	$Circumference\;ratio=\frac{C_{p}}{C_{t}}$	$Circularity=\frac{4\pi A_{p}}{(C_{p}{)}^{2}}$

*A_p_* and *C_p_* are the experimental printed area and circumference of the 3D-printed CL, respectively (calculated from the 2D top view).

**Table 2:** Curve fitting of various models for drug release kinetics.

	**Zero Order**	First Order	Higuichi Model	Korsmeyer–Peppas Model
Equation	$C_{t}=C_{0}+K_{0}t$	$\log C_{t}=\log C_{0}-(K_{1}/2.303)t$	$M_{t}/M_{\infty }=K_{h}t^{1/2}$	$\log{(M}_{t}/M_{\infty })=\log K_{kp}-n\log t$
Graph	cumulative drug release vs. time	log cumulative % drug remaining vs. time	cumulative % drug release vs. $\sqrt{t}$	log (cumulative % drug release vs. log *t*

**Table 3:** Coefficient of determination (*R*^2^) values of P-Gel-1.5% and P-Gel-3% calculated using different kinetic models.

Kinetic Model	P-Gel-1.5%	P-Gel-3%
Zero-order	0.7824	0.8131
First order	0.8487	0.9181
Higuchi	0.9364	0.9532
Korsmeyer–Peppas	0.9806	0.9864
Diffusion exponent (n-value) *	0.3464	0.3476

* Calculated by the linear regression of the Korsmeyer–Peppas equation of log (M_t_/M_∞_) versus log *t.*

## Data Availability

Data is contained within the article.
